# Pyrin Modulates the Intracellular Distribution of PSTPIP1

**DOI:** 10.1371/journal.pone.0006147

**Published:** 2009-07-07

**Authors:** Andrea L. Waite, Philip Schaner, Neil Richards, Banu Balci-Peynircioglu, Seth L. Masters, Susannah D. Brydges, Michelle Fox, Arthur Hong, Engin Yilmaz, Daniel L. Kastner, Ellis L. Reinherz, Deborah L. Gumucio

**Affiliations:** 1 Division of Radiology/Oncology, University of Alabama at Birmingham, Birmingham, Alabama, United States of America; 2 Department of Cell and Developmental Biology, University of Michigan, Ann Arbor, Michigan, United States of America; 3 Department of Medical Biology, Faculty of Medicine, Hacettepe University, Ankara, Turkey; 4 School of Biochemistry and Immunology, Trinity College, Dublin, Ireland; 5 Genetics and Genomics Branch, National Institute of Arthritis and Musculoskeletal and Skin Disease, National Institutes of Health (NIH), Bethesda, Maryland, United States of America; 6 Harvard Medical School, Laboratory of Immunology, Dana Farber Cancer Institute, Boston, Massachusetts, United States of America; University of Birmingham, United Kingdom

## Abstract

PSTPIP1 is a cytoskeleton-associated adaptor protein that links PEST-type phosphatases to their substrates. Mutations in PSTPIP1 cause PAPA syndrome (Pyogenic sterile Arthritis, Pyoderma gangrenosum, and Acne), an autoinflammatory disease. PSTPIP1 binds to pyrin and mutations in pyrin result in familial Mediterranean fever (FMF), a related autoinflammatory disorder. Since disease-associated mutations in PSTPIP1 enhance pyrin binding, PAPA syndrome and FMF are thought to share a common pathoetiology. The studies outlined here describe several new aspects of PSTPIP1 and pyrin biology. We document that PSTPIP1, which has homology to membrane-deforming BAR proteins, forms homodimers and generates membrane-associated filaments in native and transfected cells. An extended FCH (Fes-Cip4 homology) domain in PSTPIP1 is necessary and sufficient for its self-aggregation. We further show that the PSTPIP1 filament network is dependent upon an intact tubulin cytoskeleton and that the distribution of this network can be modulated by pyrin, indicating that this is a dynamic structure. Finally, we demonstrate that pyrin can recruit PSTPIP1 into aggregations (specks) of ASC, another pyrin binding protein. ASC specks are associated with inflammasome activity. PSTPIP1 molecules with PAPA-associated mutations are recruited by pyrin to ASC specks with particularly high efficiency, suggesting a unique mechanism underlying the robust inflammatory phenotype of PAPA syndrome.

## Introduction

The systemic autoinflammatory disorders include a set of heritable human diseases that are characterized by periodic attacks of fever, pain, and inflammation in the apparent absence of an infectious trigger [1]. The most common of these diseases, familial Mediterranean fever (FMF), is caused by mutations in the *MEFV* locus, which encodes the protein pyrin [2], [3]. Patients with FMF suffer sporadic and self-limited episodes of fever, accompanied by severe, localized pain [4]. Attacks involve massive neutrophil influx to affected sites, most commonly abdomen (serosal membranes), chest (pleural membranes) or joint (synovial membranes). Prophylactic treatment with colchicine, a microtubule toxin, lessens the number and intensity of attacks.

Among identified pyrin-interacting proteins is PSTPIP1 (proline serine threonine phosphatase-interacting protein 1), a cytosolic adaptor protein that functions to link PEST phosphatases to their substrates [5]. Mutations in the *PSTPIP1* gene result in PAPA Syndrome (Pyogenic sterile Arthritis, Pyoderma gangrenosum, and Acne [6], another autoinflammatory disease with severe skin and joint involvement. PSTPIP1 mutations have been shown to increase the binding affinity between PSTPIP1 and pyrin, suggesting that pyrin and PSTPIP1 are functionally linked in an unknown pathway connected to inflammation [5].

PSTPIP1 is closely related to PSTPIP2 (also called Mayp, for macrophage actin-associated tyrosine-phosphorylated protein [7], [8]). Interestingly, human PSTPIP2 maps to chromosome 18q21.3-22, the site of a susceptibility locus for chronic multifocal osteomyelitis (CRMO), another autoinflammatory disorder that affects bone and occasionally, skin and bowel [9]. Additionally, missense mutations in the murine *PSTPIP2* gene result in an osteomyelitis phenotype that is very similar to CRMO [10], [11]. Thus, both PSTPIP1 and PSTPIP2 appear to be involved in inflammatory signaling.

PSTPIP1 and PSTPIP2 share an N-terminal Fer-CIP4 homology (FCH) domain and a central coiled-coil region, through which they bind to PEST-type phosphatases [12], [13]. PSTPIP1, but not PSTPIP2, also contains a C-terminal SH3 domain that is important for the binding of several PEST phosphatase substrates, including c-abl and WASP [8], [14]. PSTPIP1, PSTPIP2 and several related proteins (including FBP-17, CIP4, Toca-1, PACSIN/syndactin/FAP52 and NOSTRIN) share not only the FCH domain, but also an extended region containing a coiled coil downstream of the FCH domain. Proteins with these shared domains comprise the EFC (extended FCH) or F-BAR (FCH-BAR) class of the BAR domain superfamily (BAR is named for Bin-Amphiphysin-Rvs). The BAR domain proteins function to link cellular membranes to the actin cytoskeleton and are involved in endocytosis [15].

Recently, it has been proposed that PSTPIP1 homo-trimerizes via its FCH and coiled-coil domain (F-BAR region) and forms a functional trimeric complex with pyrin [16]. However, the recently solved crystalline structures of several related BAR domain proteins (FCHo2, FBP17 and CIP4), reveal that these molecules form obligate dimers that are linked in a softly curved banana shape [17], [18]. The ability of these dimers to further associate into filaments is shared by several members of the F-BAR family [19], [20]. The cellular filaments formed by the PSTPIP1-related proteins FBP17 and FCHo2 are actually composed of tubular membrane-associated structures which likely represent extended endocytotic vesicles that have not undergone scission [17], [19], [20], [21]. Indeed, the F-BAR domains of both PSTPIP1 and PSTPIP2 can bind to artificial liposomes containing phosphatidyl inositol (4,5) bisphosphate (PI(4,5)P2) with high affinity [19]. Furthermore, binding induces tubulation of these liposomes in vitro [19].

In this report, we confirm that cytosolic filamentous structures formed by the PSTPIP1 protein are membrane associated and we identify the minimal domains of PSTPIP1 that are required to form these filamentous elements. We present biochemical and molecular modeling evidence that PSTPIP1 exists as a dimer and predict a cohesive pyrin-binding interface. In addition, we explore the connection between PSTPIP1 filaments and the cellular cytoskeleton and demonstrate that pyrin binding affects PSTPIP1 filament distribution. Finally, we examine the binding relationships between PSTPIP1, pyrin, and the pyrin-binding protein ASC. The results of these analyses suggest a revised model of pyrin/PSTPIP interaction in the context of inflammatory disease.

## Materials and Methods

### Plasmids and Antibodies

All FLAG-tagged constructs were cloned into pCMV-Tag2B and myc-tagged constructs were generated using pCMV-Tag3A (both from Stratagene, La Jolla, CA). The pHIS8 plasmid used for expression of PSTPIP1 in *E. coli* was previously described [22]. A QuikChange II Site-directed Mutagenesis Kit (Stratagene, La Jolla, CA) was used to generate PSTPIP1 and pyrin mutants. Fluorescent labeled constructs were expressed using pEYFP-C1 and pEGFP-C2 vectors from Clontech (Mountain View, CA). Anti-myc (rabbit polyclonal) and anti-FLAG, anti-tubulin, and anti-vimentin (all mouse monoclonal) antibodies were obtained from Sigma (St. Louis, MO). Actin filaments were visualized using AlexaFluor488 Phalloidin from Molecular Probes by Invitrogen (Eugene, OR). Fluorescent-labeled secondary antibodies (AlexaFluor488 goat anti-mouse and AlexaFluor568 goat anti-rabbit) were also obtained from Molecular Probes by Invitrogen (Eugene, OR). The PSTPIP1 (anti-CD2BP1) antibody 8C93D8 used for immunofluorescence studies [23] and the anti-human PSTPIP1 used for Western blotting[5] were previously described.

### Cell Culture and Transfection

COS-7 and HeLa cells were grown in DMEM (Gibco by Invitrogen, Carlsbad, CA) supplemented with 10% FBS (vol/vol). Cells were transfected using *FUGENE-6* (Roche Applied Science, Indianapolis, IN). Cells were fixed 24–36 hours after transfection.

### Isolation of human cells

Human blood was collected from healthy volunteers (IRB# 1992-0480) and mixed with an anti-coagulation solution containing 0.14 M anhydrous citric acid, 0.20 M citric acid trisodium salt, and 0.22 M dextrose. Red blood cells were sedimented by addition of 6% dextran and incubation for 30–45 minutes at room temperatures. The upper, leukocyte-rich layer was removed and remaining red blood cells were destroyed by hypotonic lysis in distilled water. Leukocytes were concentrated by centrifugation, and layered onto Ficoll/Paque (Pharmacia, Uppsala, Sweden) density gradient. Following centrifugation of the gradient, the neutrophil layer was removed, fixed, and stained. The monocyte/lymphocyte layer was removed, and monocytes were enriched by cell adhesion. Non-adherent lymphocytes were washed off 24 hours later, and the adherent monocytes were fixed and stained. To generate CD14+ lysates for Western blotting, CD14+ cells were enriched from PBMC populations using magnetic separation (Miltenyi).

### Immunofluorescence

Cells were fixed for 30 minutes in a 4% parafomaldehyde solution in PBS. After permeablization in 0.2% Triton-X in PBS for 10 minutes, cells were exposed to blocking solution (10% goat serum, 1% BSA, and 0.1% Tween-20 in PBS) for 1 hour. After antibody staining, 10 mM DAPI was applied to the cells for 1 minute for nuclear visualization. Coverslips were mounted with ProLong Gold Anti-Fade Reagent (Molecular Probes by Invitrogen, Eugene, OR). Slides were visualized using a Nikon E800 fluorescence microscope.

### Nocodazole Assays

Twenty-four hours after being transfected with PSTPIP1, HeLa cells were treated with 4.15 µM nocodazole and incubated on ice for 10 minutes to depolymerize microtubules. Cells were then rinsed 3 times in media and observed at 37°C in order to watch microtubules re-form.

### Cytochalasin-D Assays

A 2 mM stock of cytochalasin D was prepared in chloroform. At the time of experiment this stock solution was diluted in cell culture medium to a working concentration of 10 µM. Cells were treated for 30 minutes and then fixed with 4% paraformaldehyde in PBS.

### DiI_C16_ Membrane Staining

The DiI_C16_ membrane staining protocol was performed as previously described [21]. Twenty-four hours after COS-7 cells were transfected, cells were washed three times with DMEM without phenol red. DiI_C16_ (Invitrogen, Carlsbad, CA) was diluted to a concentration of 1.0 mg/mL in DMSO, then further diluted to 1 µg/mL in DMEM without phenol red. DiI_C16_ was applied to the cells, and cells were incubated at 37°C for 10 minutes. Cells were then rinsed two times in PBS and fixed for 30 minutes in 2% paraformaldehyde in PBS.

### PSTPIP1 structure prediction

A modeled structure of PSTPIP1 as a dimer was generated based on the structure of FBP17 (pdb accession code 2EFL) [18] using the program Swiss-Model [24]. Images and electrostatic properties of the model were generated using an implementation of APBS [25] in the program PyMOL (http://www.pymol.org).

## Results

### Comparison of PSTPIP1 and Pyrin distribution in native and transfected cells

**Figure 1:**
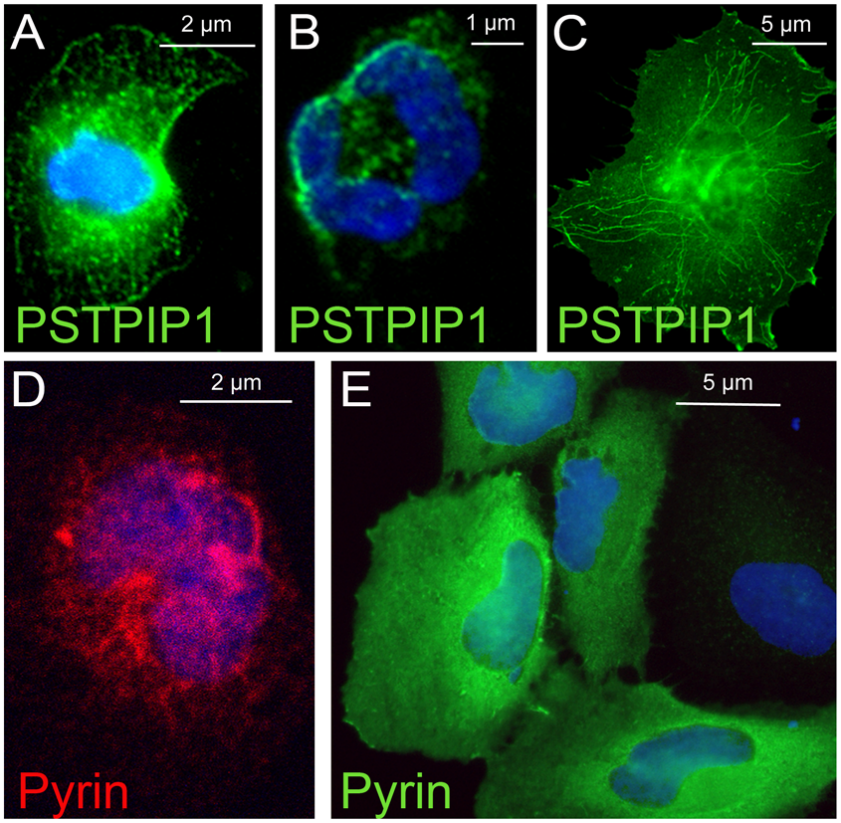
Patterns of pyrin and PSTPIP1 expression in native and transfected cells. (A) Immunostaining of native PSTPIP1 (green) in human monocytes reveals a finely branched pattern of filaments. (B) In human neutrophils, the PSTPIP1 distribution is filamentous and concentrated at the edge of the cell. (C) In transfected COS cells, PSTPIP1 forms long straight filamentous structures. (D) In human monocytes, pyrin (red) is distributed in a filamentous reticular pattern that extends throughout the cytoplasm and encircles the nucleus. (E) When epitope tagged pyrin (green) is transfected into COS cells, no reticular network is seen; pyrin is diffusely cytoplasmic.

PSTPIP1 in human monocytes appears as a branched network of fine filaments (Figure 1A). In neutrophils, filamentous staining of PSTPIP1 is suggested, but is less distinct; PSTPIP1 often appears concentrated on the cell's edge (Figure 1B). A recent study confirms that in migrating neutrophils, PSTPIP1 is polarized in the trailing edge of the uropod, the compartment that regulates both endocytosis and migration of these cells [26]. In transfected cells, which endogenously express neither pyrin nor PSTPIP1, transfected PSTPIP1 assembles into delicate filaments (Figure 1C). As previously reported, expression of PSTPIP1 results in filopodial extension in some transfected cells [27]. Unlike the extensively branched network seen in native monocytes (Figure 1A), the PSTPIP1 filaments in transfected cells are relatively straight, and seem to radiate from the nucleus to the cell periphery. Previous investigators have noted that PSTPIP1 and PSTPIP2 as well as FBP17 and CIP4 form filaments in transfected cells [19].

It has been established previously that in human monocytes, endogenous pyrin is cytosolic while in neutrophils, pyrin is nuclear [28]. We confirmed those findings and further noted that pyrin in human monocytes is distributed in a cage-like network of course filaments that surrounds the nucleus (Figure 1D). In contrast, when pyrin is transfected into cultured cells, it is uniformly distributed throughout the cytoplasm without a filamentous pattern (Figure 1E). It was not possible to simultaneously image both pyrin and PSTPIP1 in human cells since both primary antibodies were generated in rabbits.

### PSTPIP1 filament formation in transfected cells: lack of requirement for the SH3 domain and effect of PAPA-associated mutations

**Figure 2:**
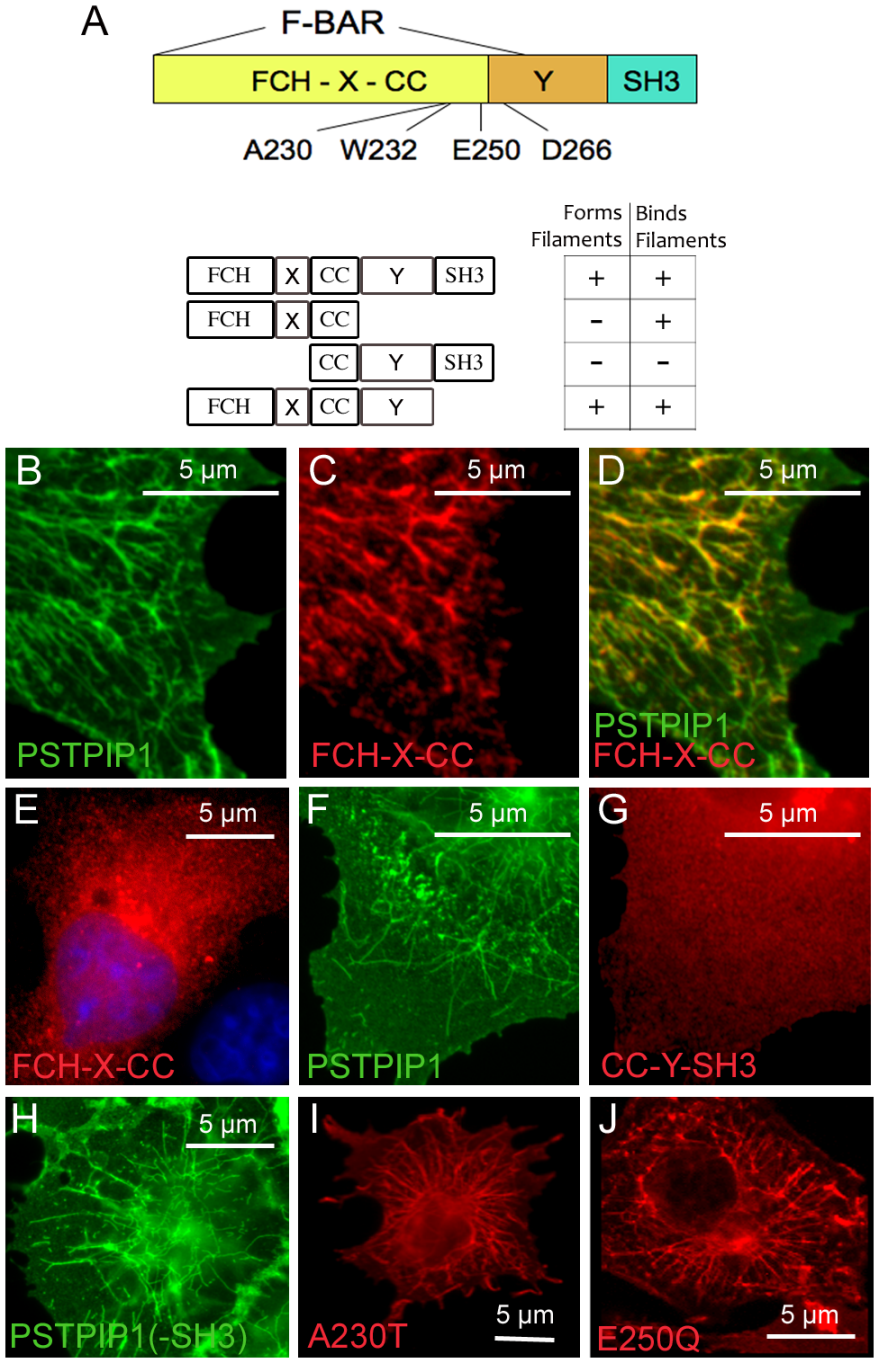
Identification of the domains of PSTPIP1 that are required for filament formation and filament binding. (A) A schematic view of the PSTPIP1 molecule (416 amino acids) is shown at top. The F-BAR domain (∼300 amino acids) includes the FCH domain, an uncharacterized intervening region (X) and a coiled coil domain (CC). A short helical region (helix 5) of the F-BAR structure is contained within the 5′ end of an uncharacterized region (Y) downstream of the CC domain [17]. The SH3 domain includes amino acid residues 364–416. The location of three mutations associated with PAPA syndrome (at amino acids 230, 250 and 266) is shown; a mutation at position 232 abolishes PSTPIP1 binding to pyrin and to PTP HSCF, a PEST-type protein tyrosine phosphatase (PTP) [5]. The specific deletion constructs tested are outlined in the lower schematic. Results of these studies are tabulated at right. “Forms filaments” indicates that the protein forms filamentous structures when transfected alone. “Binds filaments” means that the protein binds to formed full length filaments of co-transfected PSTPIP1. (B–J) Truncated versions of myc-tagged PSTPIP1 shown in (A) were transfected alone or in combination with full-length PSTPIP1-FLAG. B–E) The FCH and coiled-coil portion of PSTPIP1 bound filaments formed by full-length PSTPIP1 (B–D), but was not able to form filaments when transfected alone (E). (F,G) A PSTPIP protein containing the coiled-coil and SH3 region of PSTPIP1 was not able to form filaments (not shown), nor was it able to bind to filaments formed by full-length PSTPIP1 (G). (H) PSTPIP1 lacking the SH3 domain forms filaments when transfected alone. Thus, the SH3 domain is not required for filament formation. (I,J) The two PAPA-associated mutants, A230T (I) and E250Q (J) form long straight filaments similar to those of wildtype PSTPIP1.

The domains of the PSTPIP1 molecule and their relationship to PAPA-associated mutations are schematically shown in Figure 2A. To identify the domains of PSTPIP1 that are needed to generate the characteristic filamentous structures, we transfected COS-7 cells with full-length and/or truncated versions of epitope-tagged PSTPIP1 (Figure 2A). We tested whether each fragment was able to form filaments if transfected alone or bind to filaments when co-transfected with full length PSTPIP1. The individual cells shown in Figure 2B–J are representative of the most common staining pattern seen. The F-BAR region, encompassing the FCH and the CC, was able bind to filaments formed by full-length PSTPIP1 (Figure 2B–D). However, when it was transfected alone, this fragment did not form filaments (Figure 2E). The C-terminal CC-Y-SH3 region alone (in the absence of an intact F-BAR domain) did not form filaments (data not shown) and was unable to bind to formed filaments when co-transfected with full length PSTPIP1 (Figure 2F–G). However, the FCH-X-CC-Y fragment, lacking only the SH3 domain, was sufficient to form filaments (Figure 2H). Thus, though the SH3 domain is dispensable for filament formation, the remainder of the molecule is required. This result is fully concordant with previous findings that PSTPIP2, which lacks the SH3 domain, also forms filamentous structures readily in cells [19].

All of the known PAPA-causing mutations (A230T, E250Q, E250K, and D266N) are contained within the region of PSTPIP1 that is important for filament formation. Thus, we tested whether two of these mutations, A230T and E250Q, alter the ability of PSTPIP1 to form fibrils. Expression of flag-tagged versions of both mutant forms of PSTPIP1 resulted in filaments that were similar to those formed by wild type PSTPIP1 (Figure 2I–J).

### PSTPIP1 filaments are dependent on an intact tubulin cytoskeleton

**Figure 3:**
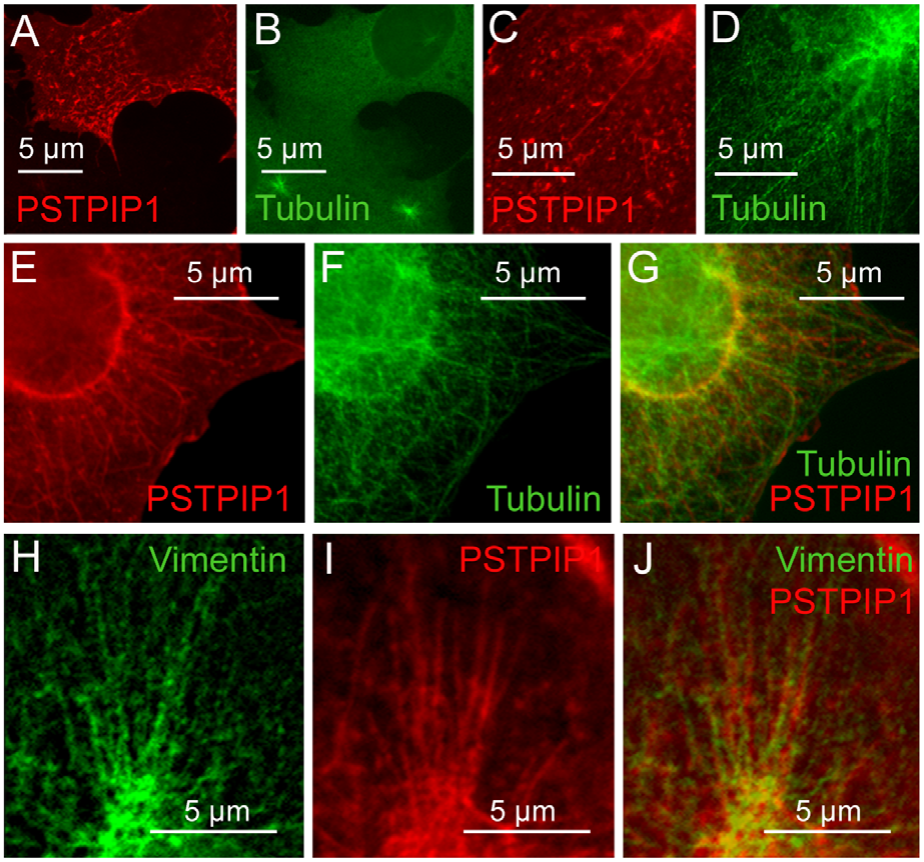
PSTPIP1 filament structure requires microtubules. Immediately after nocodazole treatment, microtubules are destroyed, as are PSTPIP1 filaments (not shown). (A–B) Within five minutes of nocodazole removal, microtubules began to rebuild, and PSTPIP1 aggregates are seen. (C–D) Thirty minutes of nocodazole washout, microtubules are reconstituted and some PSTPIP1 filaments are visible. (E–G) PSTPIP1 filaments align closely but not exactly with the tubulin cytoskeleton (H–J) PSTPIP1 filaments align next to vimentin filaments, but do not directly overlap these filaments.

The organization of PSTPIP1 filaments, with long, straight fibrils that often appear to radiate from a common origin near the nucleus, is reminiscent of the orderliness of the tubulin cytoskeleton. Indeed, the homologous F-BAR domain of CIP4 interacts with microtubules [14] and the related N-BAR family member, amphiphysin, interacts with a linker protein (CLIP-170) that binds to microtubules [29]. These data suggest that the PSTPIP1 pattern may arise from a direct or indirect association with microtubules. We used nocodazole as a reversible means of depolymerizing microtubules in transfected cells, and examined filament structure after nocodozole treatment as well as during recovery induced by nocodozole washout. Destruction of the tubulin cytoskeleton by nocodozole eliminated PSTPIP1 filaments. However, within one minute post-nocodazole washout, the microtubules began to rebuild (Figure 3A, B). As the tubulin structure was restored, a coordinated reconstruction of PSTPIP1 filaments ensued (Figure 3C, D). Both microtubules and PSTPIP1 filaments were almost completely restored by 30 minutes post-washout (Figure 3E–G) though PSTPIP1 filaments did not precisely co-localize with microtubules (Figure 3G). Similar results were obtained in the case of the N-BAR protein, amphiphysin: a filamentous distribution of amphiphysin was observed in close proximity to microtubules; these filaments were lost upon nocodozole treatment and regained after nocodozole washout [29].

Intermediate filaments (IF) also depend upon the microtubular cytoskeleton for proper cellular organization within the cell; thus IF, or IF-associated proteins may also contribute to the structure of PSTPIP1 filaments. Indeed, co-staining with vimentin revealed that PSTPIP1 filaments lie close to IF, but the two proteins do not directly co-localize (Figure 3H–J).

### Pyrin expression alters PSTPIP1 distribution

**Figure 4:**
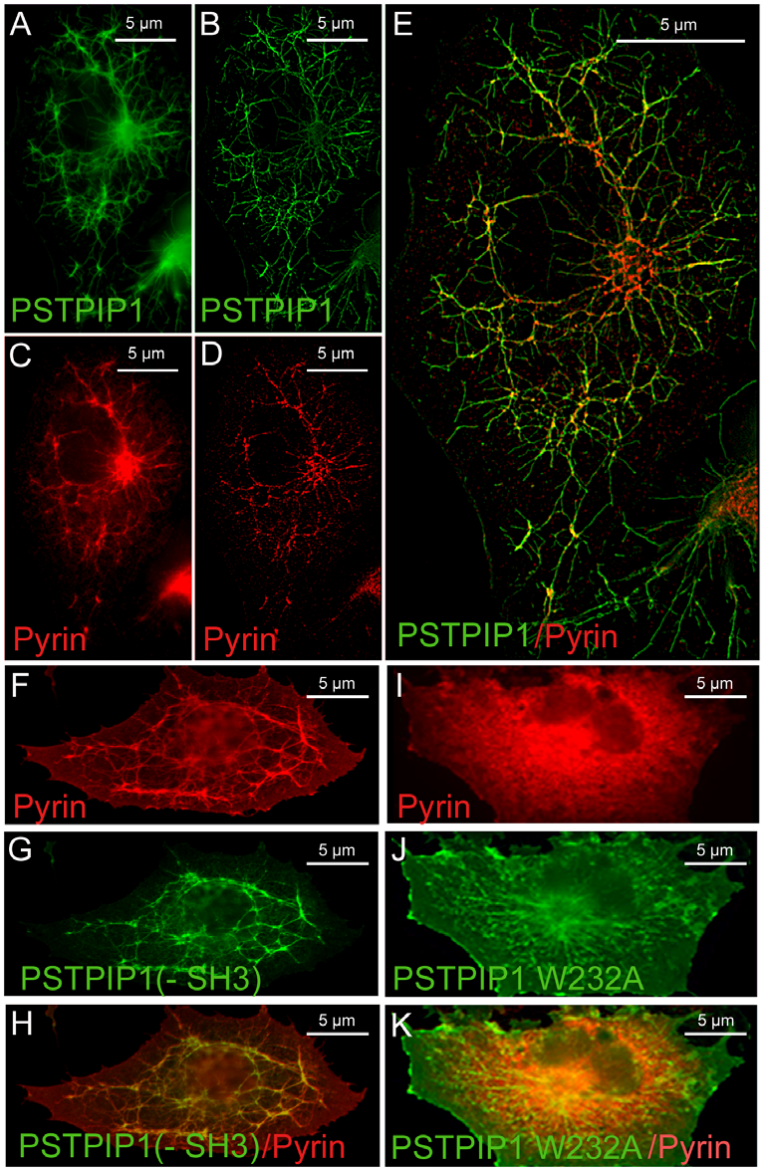
Co-expression of pyrin alters the distribution of PSTPIP1 in transfected cells. (A–E) In cells co-transfected with myc-tagged pyrin and FLAG-tagged PSTPIP1, PSTPIP1 filaments are branched and reticulated, and pyrin co-localizes with these filaments. (A) and (C) illustrate the original images, while (B) and (D) are processed images that have been deconvoluted to remove background and enhance the signal of the filaments. A branched network of filaments surrounding the nucleus is evident. (E) Overlay of pyrin and PSTPIP1 staining pattern after deconvolution; pyrin appears to be concentrated at the nodes of branch points. (F–H) FLAG-tagged PSTPIP1 lacking the SH3 domain, PSTPIP1(-SH3), can recruit myc-tagged pyrin to filaments. Pyrin binding causes the filaments to be highly branched or reticular (compare the PSTPIP1 pattern in Figure 4G and Figure 2H). (I–K) The W232A mutation of PSTPIP1, which cannot bind pyrin, forms straight filaments (green, J), and pyrin (red, I) does not decorate or reticularize these filaments. The overlay is shown in (K). Note that though filaments appear yellow, there is no filamentous pattern of pyrin (I); rather, pyrin is uniformly distributed.

Pyrin is a known PSTPIP1-interacting protein [5]. The distribution of pyrin in transfected cells that do not express PSTPIP1 is diffusely cytoplasmic (Figure 1E), but pyrin exhibits a course filamentous distribution in human monocytes that express PSTPIP1 (Figure 1D). Therefore, we tested whether pyrin would be recruited to PSTPIP1 filaments in cells co-transfected with both proteins. Indeed, this is the case (Figure 4A–E). Moreover, the long, relatively straight fibrils characteristic of transfected PSTPIP1 (Figure 1C) became branched and reticular in the presence of pyrin (Figure 4A,B). To more easily follow the course of these filaments in transfected cells, the images were de-convoluted using image processing software to remove background noise and enhance the signal from filaments. The deconvoluted images revealed a network of branching fibrils extending throughout the cytoplasm and surrounding the nucleus, with pyrin particularly concentrated at the branch points of the filaments (Figure 4C–E). Thus, pyrin binds and remodels the filamentous PSTPIP1 architecture.

### Domains of pyrin and PSTPIP1 required for pyrin recruitment and pyrin-mediated filament redistribution

The binding of PSTPIP1 and pyrin was previously studied by immunoprecipitation and the SH3 domain of PSTPIP1 was found to be necessary but not sufficient for this interaction [5]. Additionally, a mutation in the CC region of PSTPIP1, W232A, abolished PSTPIP1 binding both to pyrin [5] and to the PEST phosphatase, PTP-HSCF [8]. We therefore examined the effect of the W232A mutation and the requirement for the SH3 domain on pyrin's recruitment to and reticularization of PSTPIP1 filaments. Figure 4F–H shows that the filaments formed by PSTPIP1(-SH3) effectively recruited full length pyrin. Furthermore, the pyrin-decorated PSTPIP1(-SH3) filaments formed a branched, reticular network similar to that seen with full-length PSTPIP1. Thus, the SH3 domain is apparently dispensable for the interaction between pyrin and PSTPIP1. In contrast, the W232A mutation in PSTPIP1 abolished pyrin binding; consequently, PSTPIP1 filaments were primarily straight, not extensively branched (Figure 4I–K), confirming that the reticularization of PSTPIP1 filaments is a direct consequence of pyrin binding. Note also that PSTPIP1 filaments, in the absence of pyrin decoration, are finer and more delicate in nature (compare Figure 4G to Figure 4J)

**Figure 5:**
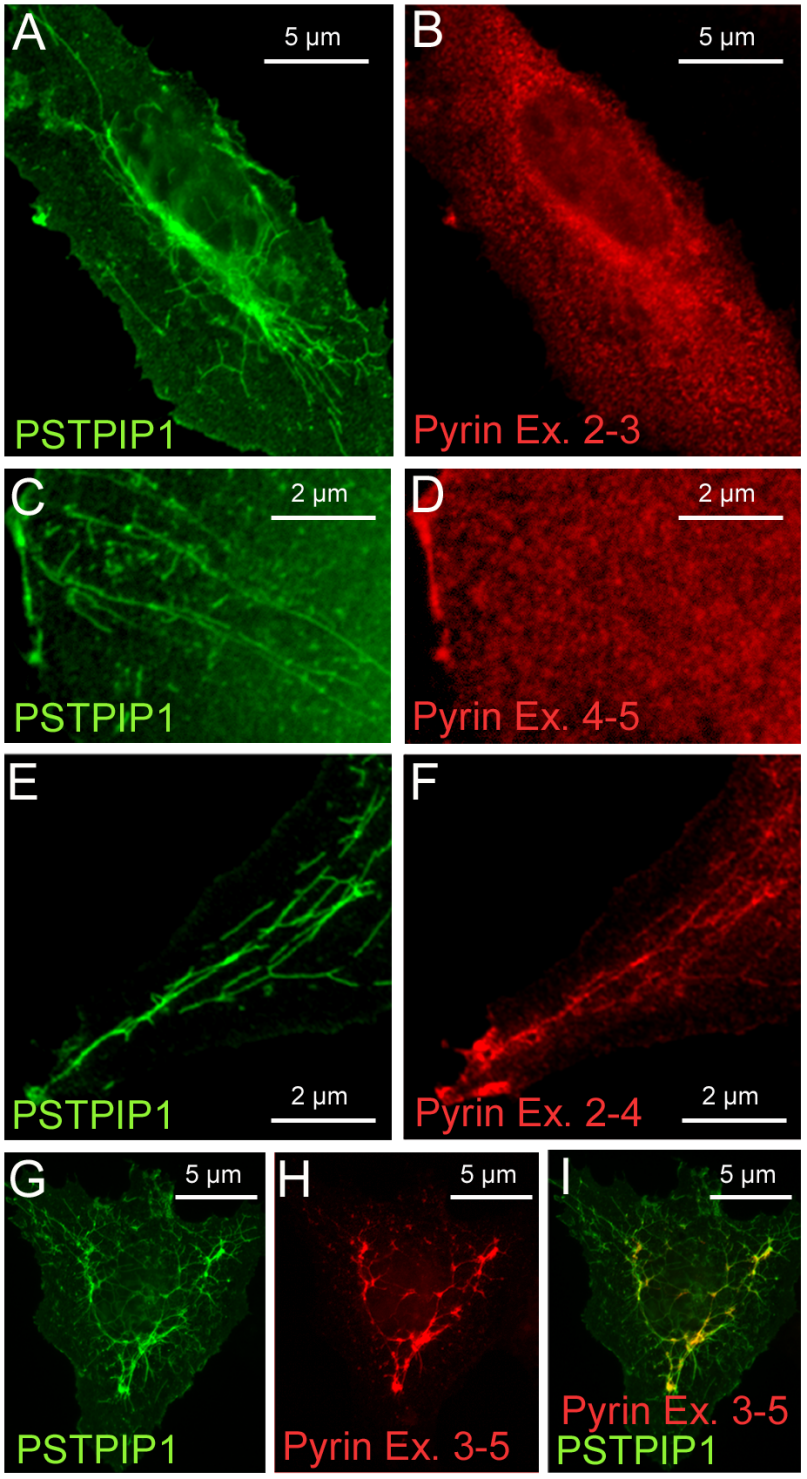
The B-box/coiled-coil region of pyrin is required for reticularization of PSTPIP1 filaments. Portions of pyrin's B-box and coiled-coil region (all myc tagged), were co-transfected with PSTPIP1-FLAG. (A–B) Pyrin exons 2–3 or (C–D) exons 4–5 do not bind PSTPIP1 filaments. Note that PSTPIP1 filaments (A,C) are generally straight. (E–F) Pyrin exons 2–4 decorates PSTPIP1 filaments, but does not alter their distribution. (G–I) The B-box and coiled-coil region of pyrin, encoded by exons 3–5, binds to and remodels PSTPIP1 filaments.

We next examined smaller fragments of the pyrin molecule to determine which regions are required for PSTPIP1 binding and filament redistribution. Pyrin exon 3 encodes the B-box motif, while exons 4–5 encode much of the coiled coil (CC) region of pyrin. Neither of these pyrin fragments bound to PSTPIP1 filaments (Figure 5A–D). Pyrin exons 2–4 produced a protein capable of aligning with PSTPIP1 filaments, but the PSTPIP1 filament network was not reticularized (Figure 5E–F). Only when the entire B-box/CC region of pyrin (exons 3–5), was expressed with PSTPIP1 did we observe a branched PSTPIP1 filament pattern similar to that induced by full-length pyrin (Figure 5G–I); pyrin exons 3–5 co-localized with this filamentous PSTPIP1 network.

### Mutations in pyrin or PSTPIP1 do not alter the pattern of PSTPIP1 filaments

**Figure 6:**
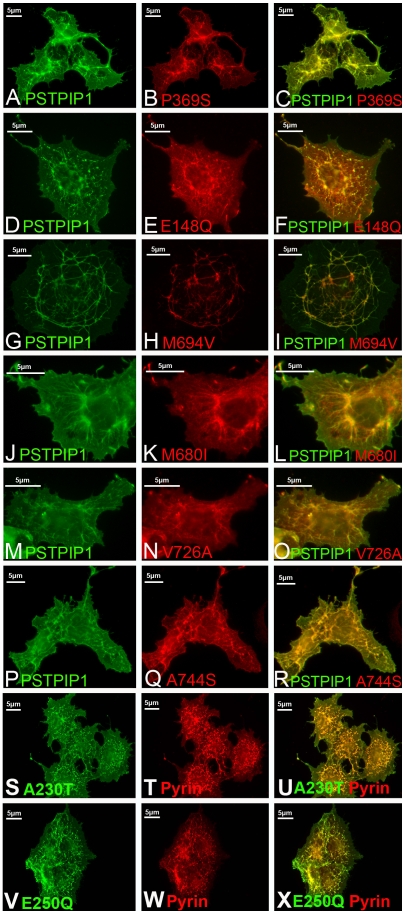
FMF-causing mutations do not alter the appearance of reticular PSTPIP1 fibrils; PAPA-associated PSTPIP1 mutants are bound and reticulated by pyrin. (A–R) Myc-tagged versions of mutant pyrin were co-transfected with PSTPIP1-FLAG: (A–C) P369S; (D–F); E148Q; (G–I) M694V; (J–L) M680I; (M–O) V726A; (P–R) A744S. (S–X) PAPA syndrome-associated PSTPIP1 were co-transfected myc-pyrin: (S–U) A230T; (V–X) E250Q.

It has been proposed that pyrin and PSTPIP1 are functionally linked in an unknown pathway that is connected to the innate immune system [5], [16]. We therefore tested whether FMF-causing mutations in pyrin would modulate the interaction between these two proteins or alter the distribution of PSTPIP1 filaments. Myc-tagged versions of pyrin mutants were co-transfected with FLAG-tagged PSTPIP1 in COS-7 cells. We tested pyrin mutations that are located in exon 2 (E148Q), the B-box region (D330A, P369S), the coiled coil region (R408Q, E474K, H478K, F479L) and the B30.2/SPRY domain (V726A, A744S, M680I, M694V, Y688X). All pyrin mutants bound to PSTPIP1 and reticularized PSTPIP1 filaments in a manner similar to wildtype pyrin (Figure 6A–R). Similarly, in previous studies using immunoprecipitation, mutations in pyrin did not modify its interaction with PSTPIP1 [5].

To explore the effect of PAPA mutations on the PSTPIP1:pyrin interaction, we next tested whether the filaments formed by mutant forms of PSTPIP1 could be reorganized by pyrin. We found that the PAPA-causing mutations, A230T and E250Q (Figure 6S–X), were reticularized by pyrin in a manner similar to that seen with wildtype PSTPIP1. We conclude that disease-causing mutations in either pyrin or PSTPIP1 do not alter the basic reticular nature of the pattern seen with co-expression of the wildtype proteins, though alterations in the function of the filament system cannot be ruled out.

### PSTPIP1 filaments are lipid membrane associated

**Figure 7:**
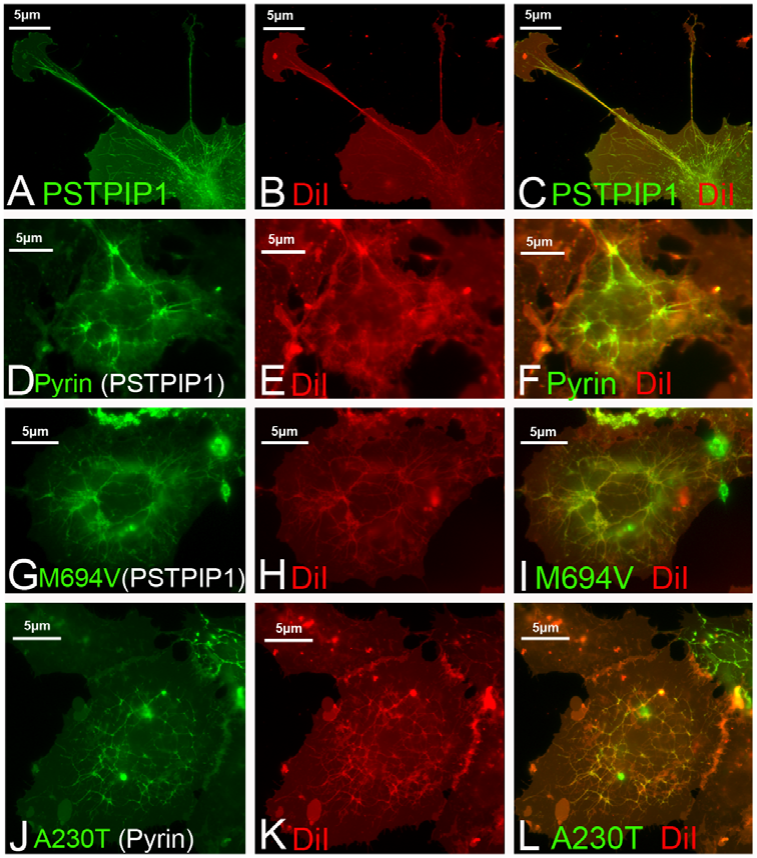
PSTPIP1 filaments are membrane-associated. (A–C) DiI_C16_ decorates PSTPIP1 filaments, indicating that the fibrils are membrane-associated. (D–F) In cells transfected with pyrin and PSTPIP1, pyrin produces the typical reticular pattern, and DiI_C16_ staining is associated with the branched filaments. (G–I) In cells transfected with pyrin M694V-YFP and wild type PSTPIP1, filaments are visible and stain with DiI_C16_. (J–L) In cells transfected with wild type pyrin and PAPA-associated A230T-GFP, DiIC_16_ filament staining is preserved.

PSTPIP1 is a member of the F-BAR family of proteins [19]. These proteins are known for their ability to bind to and bend cellular membranes [30], [31], a property that can be revealed by staining with lipophilic dyes such as DiI_C16_ [21]. To assess whether PSTPIP1 filaments are membrane associated, we transfected COS-7 cells with GFP-tagged PSTPIP1 and then exposed the cells to DiI_C16_, a fluorescent lipophilic dye that associates with cellular membranes. PSTPIP1 filaments co-localized with DiI_C16_ staining (Figure 7A–C) as previously described for the related protein, FBP17[21]. In the presence of pyrin, DiI_C16_ staining was not perturbed (Figure 7D–F). Finally, mutations in pyrin (Figure 7G–I) or in PSTPIP1 (Figure 7J–L) did not affect DiI_C16_ staining. Taken together, these data confirm that PSTPIP1 shares the lipid membrane-binding properties of several other F-BAR domain-containing proteins, a property that is not affected by pyrin binding.

### Modeling PSTPIP1 structure: prediction of separate membrane-interacting and pyrin-interacting surfaces

**Figure 8:**
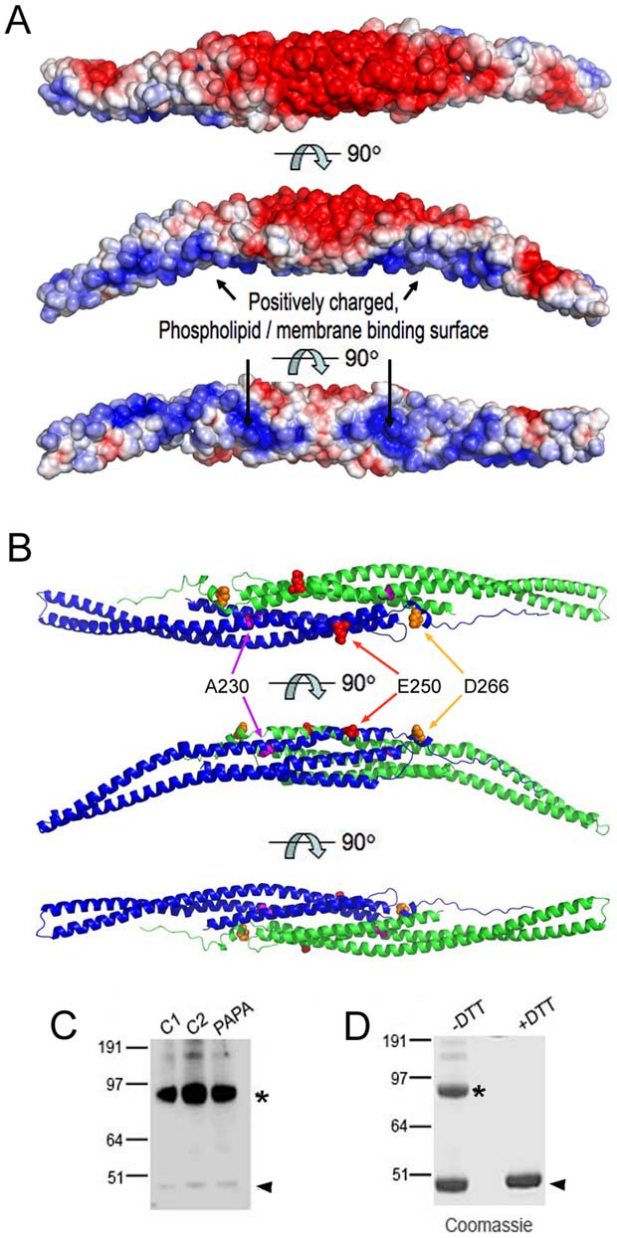
Molecular modeling of PSTPIP1 reveals a banana-shaped dimer and independent coherent binding faces for lipid membranes and for pyrin. The structure of the PSTPIP1 FCH domain was generated, based on that of FBP17. (A) A space-filled representation of a PSTPIP1 dimer is presented, with solvent accessible charges highlighted in blue (positively charged) and red (negatively charged). The three representations present three surfaces, as the dimer is rotated by 90° around the x-axis. A positively charged surface is accessible on the concave surface of the domain, similar to that seen in FBP17 and FCHo2; this surface has been determined experimentally in those molecules to be the lipid membrane binding face. (B) In a ribbon diagram of the PSTPIP1 dimer, one molecule is colored green, while the other is blue. Mutations that cause PAPA syndrome, indicated in three different colors, group together on the convex surface of the molecule. This cohesive face, opposite the membrane binding face, likely mediates binding interactions with pyrin and PTP-PEST proteins. (C) Western blot CD14+ lymphocyte lysates from controls (C1, C2) and a PAPA patient (PAPA) in non-reducing conditions, probed with anti-PSTPIP1 reveal a major species at 96 kDa (*), corresponding to dimerized PSTPIP1. Small amounts of monomer (48 kDA, arrowhead) and multimer (trimer and tetramer, bands at top of gel) are also visible. (D) Recombinant human PSTPIP1 protein expressed in E. coli is stained with Coomassie reagent. The 98 kDa dimer (*) is prominent when the protein is run in the absence of reducing agent, while in the presence of DTT, a 48 kDa monomer (arrowhead) is exclusively seen. Since multiple steps in the recovery of the recombinant protein require addition of DTT, some monomer is present even when additional reducing agent is not added for the electrophoresis (Lane 1).

We generated a model of PSTPIP1 molecular structure, based on the published structure of the FBP17 dimer [18]. Predicted PSTPIP1 dimers contained a similar gently curved surface. Predicted solvent accessible positively charged residues appear on the concave side (Figure 8A), the region that has been predicted to interact with the phospholipid membrane. The charge distribution in the modeled PSTPIP1 molecule is similar to that seen in other BAR domain proteins [17], [18], and implies a similar mechanism for membrane interaction and filament formation.

We next used the modeled ribbon structure of PSTPIP1 to predict the location of the PAPA-associated mutations. As shown in Figure 8B, all three of these amino acid residues (A230, E250 and D266) are located on the convex side of the molecule. This predicts that these mutations would not alter the ability of the protein to interact with lipid membranes on its concave surface, a prediction that is borne out experimentally (see Figure 7). The model also suggests that the coherent convex binding surface would likely interact with both pyrin and the PEST phosphatases, since these PSTPIP1 mutations have been shown to reduce binding affinity for PEST phosphatases and increase affinity for pyrin (Shoham, 2005).

### Endogenous PSTPIP1 forms dimers

Computer modeling results presented above suggest that PSTPIP1 exists as a dimer. However, other studies have suggested that the major form of PSTPIP1 is a trimer [16]. To examine the state of PSTPIP1 in native cells, we probed Western blots of CD14+ cell lysates from control individuals and a PAPA syndrome patient for PSTPIP1 (Figure 8C). In all three cases, the majority of the PSTPIP1 signal was seen as a 95 kDa species, corresponding to a PSTPIP1 dimer. A minor species at 48 kDa corresponded to monomeric PSTPIP1 while faint multimeric forms of PSTPIP1, likely trimers and tetramers, were also observed. Recombinant PSTPIP1 protein was also generated and examined in Western blots in the presence or absence of reducing agents (DTT). While unreduced PSTPIP1 protein ran as a dimer as well as a monomer, only the monomer form was seen in the presence of DTT (Figure 8D). Taken together, these results indicate PSTPIP1 exists mainly as a dimer, consistent with the molecular modeling predictions and consistent with data for related BAR proteins [17], [18].

### Interactions among PSTPIP1, ASC and pyrin; effect of mutations in PSTPIP1 or pyrin

Pyrin can interact with both PSTPIP1 [5] and ASC [32]. Pyrin's N-terminal pyrin domain facilitates the latter interaction while pyrin's B-Box/coiled coil mediates the former. Current published evidence favors a model of ASC/PSTPIP1/pyrin interaction in which normally autoinhibited pyrin molecules require PSTPIP1 mediated unfolding in order to interact with ASC [16]. If this is the case, then pyrin and ASC should only co-localize in the presence of PSTPIP1.

**Figure 9:**
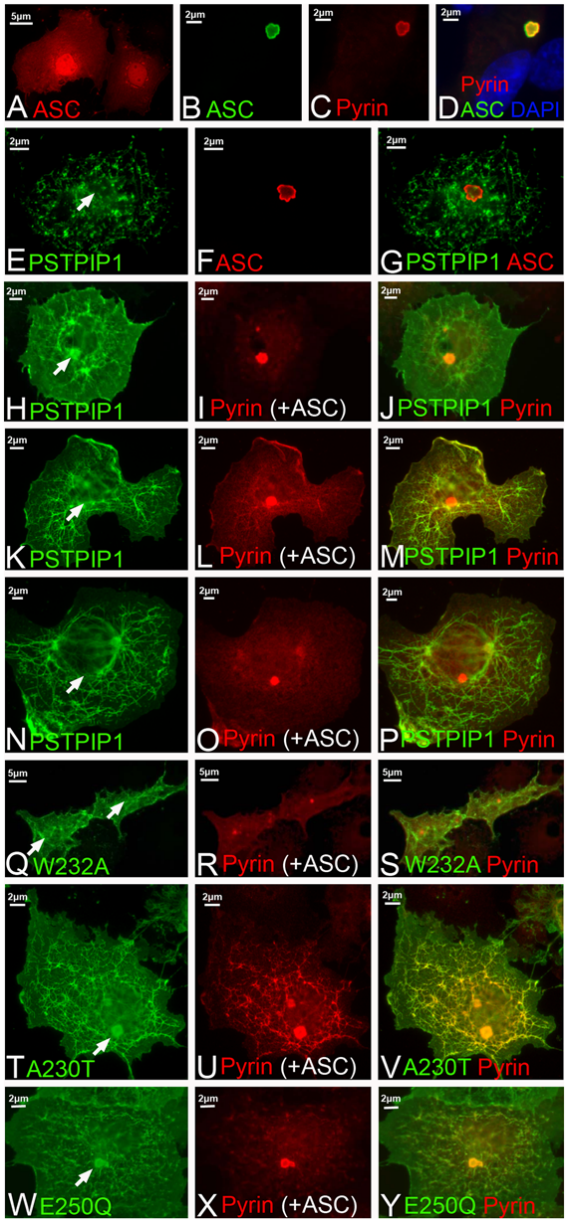
Pyrin recruits PSTPIP1 to ASC specks. All images are from transfected COS cells. Representative images are shown. (A) The apoptotic speck protein, ASC (red), is normally diffusely distributed throughout the cell in cytoplasm and nucleus. (B) ASC (in this case, green) can coalesce into a small perinuclear aggregate, the speck. (C–D) Pyrin (red) is recruited to ASC specks via its PyD as previously shown [32]. (E–G) PSTPIP1 is not detected in ASC specks in the absence of co-transfected pyrin. (H–J) In 70% of cells transfected with untagged ASC, PSTPIP1-FLAG and pyrin-myc, PSTPIP1 is recruited to the speck (arrow, H). (K–P) In 30% of cases, transfection of the three proteins results in localization of pyrin in both PSTPIP1 filaments and in the speck (K–M), or exclusively in the speck (N–P). (Q–S) FLAG-tagged W232A PSTPIP1 does not interact with pyrin, and is not recruited to specks. (T–Y) Recruitment of PAPA mutants by myc-pyrin to the ASC speck. (T–V) A230T-FLAG. (W–Y) E250Q-FLAG. Pyrin recruits these mutant forms to ASC specks in 95% of transfected cells.

To test this model, we transfected cells with various combinations of pyrin, ASC and PSTPIP1. When ASC specks form in cells transfected with ASC and pyrin, both proteins are always co-localized in the specks (Figure 9A–D). Thus, pyrin does not require PSTPIP1 for recruitment to the speck inflammasome compartment. Moreover, in cells transfected with PSTPIP1 and ASC, PSTPIP1 is never observed in specks (Figure 9E–G), indicating that PSTPIP1 never visits the inflammasome in the absence of pyrin. When PSTPIP1, pyrin and ASC are all co-transfected, all three proteins are co-localized in speck-like structures in 70% of cells (Figure 9H–J). Thus, pyrin apparently recruits PSTPIP1 to the speck compartment. In the remaining cells, PSTPIP1 is not associated with specks (Figure 9K–P). In these latter cases, pyrin is observed either in association with both specks and PSTPIP1 filaments (Figure 9K–M) or exclusively in ASC specks (Figure 7N–P). In no case does pyrin decorate PSTPIP1 filaments to the exclusion of the speck compartment.

We also transfected cells with pyrin-myc, untagged ASC and a FLAG-tagged version of the W232A PSTPIP1 mutant that does not bind pyrin. In this case, pyrin is localized in 100% of specks, but PSTPIP1 is never localized in specks (Figure 9Q–S). Together, these data establish that pyrin interacts more readily with ASC than with PSTPIP1 and that recruitment of PSTPIP1 to the speck compartment is pyrin-dependent. The data do not support a PSTPIP1-mediated delivery of pyrin to the ASC speck compartment.

When PAPA syndrome-causing mutants of PSTPIP1 were transfected with ASC and pyrin (A230T Figure 9T–V; E250Q Figure 9W–Y), PSTPIP1 appeared in the speck with pyrin over 95% of the time (compared to 70% of the time with wild type PSTPIP1). This corroborates earlier reports that mutant forms of PSTPIP1 bind to pyrin with higher affinity [5], and confirms that PSTPIP1 recruitment to the speck compartment depends on the PSTPIP1-pyrin interaction. Importantly, these data together indicate that mutant PSTPIP1 is recruited to an inflammasome compartment (the ASC speck, [33]) with greater efficiency than is wild type PSTPIP1. In contrast, the use of mutant forms of pyrin did not alter the frequency of recruitment of PSTPIP1 to the ASC speck (data not shown), though as shown in earlier studies, mutant pyrin does increase the rate of speck formation [32], [34].

## Discussion

The data presented here provide new insight into the biology of PSTPIP1, the molecule mutated in PAPA syndrome, and clarify the nature of its interaction with pyrin, the protein mutated in familial Mediterranean fever. This analysis indicates that an extended region of the F-BAR domain, but not the SH3 domain, is necessary for PSTPIP1 filament formation, consistent with predictions based on the F-BAR structure [18]. Though PSTPIP1 filaments do not appear to co-localize directly with intermediate filaments or microtubules, we find that the integrity of PSTPIP1 filaments depends on an intact microtubular system. We also examine the pyrin/PSTPIP1 interaction and show that contrary to earlier predictions [5], the PSTPIP1 SH3 domain is not required for its interaction with pyrin. Modeling studies support these experimental results and predict a coherent surface on the PSTPIP1 dimer that may interact with pyrin. In support of modeling studies, PSTPIP1 exists mainly as a dimeric species *in vivo*. In the cell, we show that the presence of pyrin alters the conformation of the PSTPIP1 filamentous network. Finally, we directly test the binding relationships between PSTPIP1, pyrin, and the pyrin-binding protein ASC. We find that the balance of these interactions is altered by disease-causing mutations in PSTPIP1, but not by FMF-associated mutations in pyrin. Together, the data predict that the PAPA-associated mutations may cause an altered cellular distribution of PSTPIP1 in a manner that has the potential to directly impact inflammatory signaling.

PSTPIP1 is a member of the F-BAR family of proteins, a family that also includes FCHo2, FBP17 and CIP4, among others [17], [18]. These proteins can bind and tubulate phospholipid membranes, forming a network of tubular filamentous structures in the cell. The F-BAR proteins function to couple membrane deformation to actin cytoskeletal polymerization during endocytosis [19]. We show here that pyrin can not only bind to these filamentous, PSTPIP-coated membrane tubules, but can also alter their distribution. It remains to be determined what functional consequences for the cell accompany this reticularization of the filamentous pattern (e.g., alterations in endocytosis, phagocytosis, synapse formation, migration or other processes that involve membrane deformation). We were not able to discern an effect of either pyrin mutations or PSTPIP1 mutations on the property of PSTPIP1 filament reticularization.

Except for the C-terminal SH3 domain, the entire remaining PSTPIP molecule (FCH-X-CC-Y) is required for filament formation. In the presence of full length PSTPIP1, the protein containing only the F-BAR domain (FCH-X-CC) is able to bind to formed filaments, but the uncharacterized “Y” region between the coiled coil and the SH3 domain appears to be additionally required for the generation of filaments (Figure 2). Interestingly, Henne et al. noted that, in the crystal structure of FCHo2, an extended region downstream of the coiled coil forms an alpha helix (helix 5) that is required for stabilization of the dimers [17]. This region is highly conserved between FCHo2 and PSTPIP1, and is also clearly present in the homology modeled structure of PSTPIP1; in fact, it contains residue D266 that is mutated in PAPA syndrome.

Several F-BAR proteins, including CIP4, FBP17 [18] and FCHo2 [17], have been crystallized and their structures reveal important clues about their biological properties. First, it is clear from these structures that F-BAR proteins exist as dimers that are molded in a gently curved, banana-like shape. Dimerization is likely constitutive, since the monomers contain extended dimerization faces that are stabilized by several conserved amino acid residues [18]. The lesser curvature of the dimer contains a number of positively charged patches that have been shown to bind to phospholipids. Alignment of the F-BAR domains of PSTPIP1 and PSTPIP2 with those of the other F-BAR proteins reveals a high degree of conservation. Indeed, all of the previously identified amino acids that function in dimerization are also present in the two PSTPIP proteins [17], [18], [35]. Moreover, the positively charged patches that have been shown to bind to phospholipids are also conserved [17], [18], [35] and as we demonstrate here, the PSTPIP1 F-BAR structure is easily fitted to the established crystal structure of FBP17. In the model, the PAPA-associated mutations are located on the outer (lesser curvature) face, in a position that predicts a coherent pyrin interaction surface. This region might also be a PEST phosphatase interaction surface, since pyrin and PEST phosphatase are thought to bind to the same region of PSTPIP1 and the PAPA mutations increase affinity for pyrin while decreasing affinity for PEST phophatases [5].

Like other F-BAR proteins, we show here that PSTPIP1 is connected in a dynamic way to both the cytoskeleton and to the plasma membrane. PSTPIP1 has previously been linked to the actin cytoskeleton [13], and we report an additional requirement for the tubulin cytoskeleton in generation of PSTPIP1 filamentous structure. We found that the PSTPIP1 filament structure is rapidly regenerated as the microtubular structure is re-built following nocodozole treatment and washout. The need for intact MT in endocytosis is well-established [36] and the filamentous membrane-associated tubules generated by PSTPIP1 and other F-BAR proteins are thought to reflect active endocytosis [37]. Thus, the rapid re-assembly of PSTPIP1 filaments after nocodozole wash-out may reflect re-activation of the endocytotic process. It will be important to determine whether PSTPIP1 binds directly to microtubules, like the related F-BAR protein, CIP4 [14], or whether the interaction is via an intermediate microtubule binding protein, as in the case of amphiphysin [29].

It has been proposed that pyrin and PSTPIP1 are involved in the same inflammatory signaling pathway and earlier studies suggest mechanistic pathways for their interaction. First, Shoham et al. presented a model in which PSTPIP1 binds and sequesters pyrin and showed that this interaction requires the SH3 domain of PSTPIP1 [5]. Further, this model posits that sequestration of pyrin by PSTPIP1 could hamper its ability to interact with, and inhibit, the ASC inflammasome. Yu, et al. further refined this model, presenting biochemical evidence that both pyrin and PSTPIP1 exist as homotrimers [16]. The pyrin homotrimer, they propose, is autoinhibited by the association of its pyrin domain (PyD) with its own B-box. PSTPIP1, particularly the PAPA-associated mutant forms of this protein which bind pyrin with high affinity, bind to pyrin's B-box, unmasking the PyD and allowing its interaction with the PyD of ASC. This, in turn, leads to multimerization of ASC and subsequent recruitment and activation of Caspase-1. In this model, then, PSTPIP1 is required for pyrin's functional interaction with the ASC inflammasome.

Our data suggest a model that differs from these previous models in three ways. First, our studies show clearly that the SH3 domain of PSTPIP1 is not required for pyrin binding nor is it necessary for pyrin-mediated reticularization of PSTPIP1 filaments. This difference with previous data may be related to the difficulty of accurately performing immunoprecipitations with these proteins, all of which have a high tendency to aggregate, making redissolution of the immunoprecipitated pellet difficult [32]. Structural modeling confirms the experimental findings and suggests a coherent face on the F-BAR region of PSTPIP1 (apart from the SH3 domain) that could act as a pyrin-interacting and likely PEST phosphatase-interacting surface. Based on these findings, it is tempting to speculate that the PSTPIP1-related protein, PSTPIP2, which lacks an SH3 domain, but also forms filaments in cells, might also interact with pyrin; PSTPIP2 has already been shown to interact with PEST phosphatases [8]. This will be an interesting association to test, since PSTPIP2 is also linked to inflammatory disease [9], [11].

A second difference between our findings and those of earlier studies is that we find that pyrin is not sequestered away from ASC by its interaction by PSTPIP1, and does not require PSTPIP1 to interact with ASC. Rather, pyrin interacts readily with ASC both in the presence or absence of PSTPIP1. Indeed, in earlier studies, we demonstrated that, in HeLa cells (which do not express PSTPIP1) co-transfection of pyrin and ASC promotes ASC speck formation [32].

Third, and most importantly, in the presence of ASC, pyrin recruits PSTPIP1 to the ASC speck compartment, a compartment that PSTPIP1 never visits in the absence of pyrin. Mutant PSTPIP1, by virtue of its increased binding affinity for pyrin, may be somewhat more readily recruited to ASC specks than is wild type PSTPIP1, placing mutant PSTPIP1 more frequently in the inflammasome compartment. In contrast, we find no evidence that mutations in pyrin alter the cellular distribution of PSTPIP1 in a manner that differs from wild type pyrin. Taken together, our data support the notion that the increased pyrin binding affinity of PAPA mutations alter the cellular localization of PSTPIP1, bringing it to a compartment in which it might act to intensify inflammasome signaling. Indeed, increased inflammasome activity is implied by the finding that myeloid cells from PAPA syndrome patients secrete very high quantities of IL-1β [5]. It is interesting to speculate that a similar model could also apply to the PSTPIP1-related protein, PSTPIP2. The common structures of the F-BAR domains of these two PSTPIP proteins [19] and the location of the likely binding site for pyrin within the F-BAR domain documented here predict that pyrin may also interact with PSTPIP2. If so, this might suggest that pyrin, a known regulator of inflammasome function, is etiologically linked to the pathogenesis of inflammatory diseases caused by mutant forms of both PSTPIP1 and PSTPIP2.
